# Outcome and management of children with chest indrawing pneumonia at primary health care settings in Pakistan: an observational cohort study

**DOI:** 10.7189/jogh.15.04096

**Published:** 2025-03-28

**Authors:** Zamir Hussain Suhag, Ashlesha Pal, Muhammad Naeem, Imran Ahmed, Noorulain Altaf Khuwaja, Shayan Khakwani, Ali Mujtaba, Shamim Ahmad Qazi, Yasir Bin Nisar

**Affiliations:** 1 Research Department, Trust for Vaccines & Immunization, Karachi, Pakistan; 2 Department of Pathology and Laboratory Medicine, The Aga Khan University, Karachi, Pakistan; 3 Centre of Excellence for Women and Child Health, The Aga Khan University, Karachi, Pakistan; 4 Independent consultant, Geneva, Switzerland; 5 Department of Maternal, Newborn, Child and Adolescent Health and Ageing, World Health Organization, Geneva, Switzerland

## Abstract

**Background:**

Pneumonia is a major cause of childhood mortality in Pakistan. In 2019, the Government of Pakistan revised the national Integrated Management of Childhood Illness (IMCI) chart booklet, following the World Health Organization’s recommendation for outpatient management with oral antibiotics of children aged 2–59 months with chest indrawing pneumonia. We aimed to assess the outcomes of children aged 2–59 months with chest indrawing pneumonia in programme setting of Pakistan.

**Methods:**

This was a prospective observational cohort study in three primary health care facilities in Thatta district, Sindh province. We screened children aged 2–59 months who presented with cough and/or difficult breathing, and recruited those classified with chest indrawing pneumonia according to the IMCI tool. from December 2022 to March 2024. The primary outcome was to assess the case fatality ratio. The secondary outcomes were to calculate the prevalence of antibiotic use, hospital admissions and treatment adherence among these children during the current illness. We followed up on day 15 after enrolment to assess outcomes.

**Results:**

456 children with chest indrawing pneumonia met the study’s enrolment criteria. Two (0.4%) died. Four (0.9%) were lost to follow-up and excluded from the analysis. According to parental/caregiver reports, among 452 children followed up on day 15, 435 (96.3%) were cured, 12 (2.7%) did not improve and two (0.4%) worsened and were hospitalised. All patients were treated with oral antibiotics. Oral amoxicillin was prescribed and used by 282 (62.4%) and 236 of those (83.7%) adhered to five or more days of oral amoxicillin treatment. Oral cefixime was prescribed and used by 114 children (25.2%).

**Conclusions:**

Our findings support using the IMCI protocol for treating chest indrawing pneumonia without danger signs in children aged 2–59 months with oral antibiotics on an outpatient basis. It can potentially reduce childhood pneumonia deaths, increase access to treatment, improve treatment coverage, reduce referrals and reduce costs for the health system and families in resource-limited settings.

**Registration:**

ISRCTN: 12687253.

Despite a notable worldwide decline in under-five mortality to 37 deaths per 1000 live births in 2022 [1], childhood pneumonia remains a major global health concern, responsible for over 700 000 deaths annually, and this represents 14% of deaths in this age group worldwide. The greatest incidence of pneumonia is seen in South Asia, West Africa, and Central Africa, with 2500 and 1620 cases per 100 000 children, with slow progress in reducing pneumonia-related deaths in children under five years of age [2]. The current under-five mortality rate in Pakistan stands at 61 deaths per 1000 live births [3]. Pneumonia remains a major contributor to this mortality rate, with seven deaths per 1000 live births [4], reflecting the high burden and severity of pneumonia, particularly in rural areas [5,6].

Evidence from resource-limited settings indicates that children with chest indrawing pneumonia without comorbidities, severe malnutrition, or severe pallor can be successfully treated in outpatient settings [7], particularly in low- and middle-income countries (LMICs). The World Health Organization (WHO) revised its childhood pneumonia management guidelines in 2012, recommending outpatient treatment with oral amoxicillin for children aged 2–59 months with chest indrawing pneumonia [8,9]. Subsequently, to incorporate this recommendation, WHO updated the Integrated Management of Childhood Illness (IMCI) chart booklet in 2014 [10]. This recommendation was predicted to be more cost-effective than the previous inpatient recommendation for these children. It was estimated that across the 74 countdown LMICs, if fully implemented, the updated guidelines could potentially save up to 39.8 million disability-adjusted life years and would do so at a lower cost [11]. The revised pneumonia guidelines have the potential to reduce the burden on the health system and increase access to care with lower costs, ultimately benefiting both the health system and the affected families [12].

The government of Pakistan revised the national IMCI chart booklet and training materials in 2019 [13]. However, little is known about the success of the new guidelines and their implementation challenges in real-world programme settings. We aimed to evaluate the survival status and outcomes with outpatient management of chest indrawing pneumonia in children aged 2–59 months at primary health care (PHC) facilities in a programme setting. In this context, we aimed to address the following research question: ‘What are the treatment outcomes and case fatality rates among children aged 2–59 months assessed, classified, and managed according to the IMCI tool?’

## METHODS

### Study design and setting

**Figure 1:**
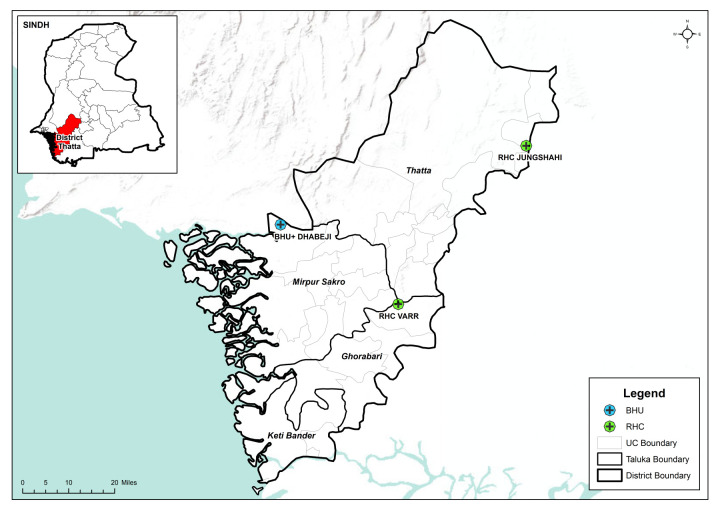
Map showing the selected primary health care facilities in Thatta district.

We conducted a prospective observational cohort study in a programme setting in Thatta district, Sindh, Pakistan, a rural district with approximately one million population [14]. We comprehensively assessed six PHC facilities using various criteria such as the availability of routine health care services, human resources, availability of adequate medicine supply, provision of routine outpatient care services – including maternal, newborn, and child health – routine immunisation, and the catchment population of each PHC facility (ranging from 15 000–30 000). Additionally, we extracted six months of data from the District Health Information System from May–October 2022. Based on the above assessments and information, we selected three PHC facilities, namely, basic health unit Dhabeji, rural health centre Varr, and rural health centre Jungshahi (**Figure 1**) [15]. The study protocol published elsewhere describes more details of the methodology [16].

### Participants

We considered eligible for study participation children aged 2–59 months with cough and/or difficult breathing, presenting at the selected PHC facilities with lower chest indrawing, and who resided in the study catchment area where follow-up at day 15 could be achieved. Children were excluded if they had any general danger signs (convulsions, inability to drink or feed, vomiting everything, lethargy or unconsciousness), oxygen saturation level (SpO_2_) < 90%, stridor when calm, or were currently enrolled in any other clinical study.

### Study procedures

#### Preparatory phase

We obtained approvals from the Department of Health, Government of Sindh, and the District Health Administration. We developed the study case report forms in English, which we later translated into the local language. We established an electronic database application to facilitate real-time data management at all study sites.

At the commencement of the study, a three-day refresher training session on the IMCI chart booklet related to the pneumonia management protocol was conducted by IMCI master trainers for selected facility-based health care providers (HCPs) to equip them with the skills to accurately assess, classify and manage pneumonia. HCPs were trained on the assessment by measuring respiratory rate and chest indrawing and identifying danger signs to accurately classify fast breathing, chest indrawing, or severe pneumonia. Based on the classification, providers learn to decide on appropriate management, such as administering the correct antibiotics (*e.g.* amoxicillin) and referrals for severe cases. This was followed by a comprehensive three-day training for the data collectors. Training covered the study tools, anthropometric measurement, use of handheld devices, and data collection procedures. We conducted a pilot study on 93 children presenting to the PHC with cough and/or difficult breathing, to validate the study tools, the data collection application, and the feasibility of the study.

#### Screening and enrolment procedures

The HCPs screened all children aged 2–59 months presenting with cough and/or difficult breathing at the selected PHC facility (day 1) using the IMCI chart booklet to assess, classify, and manage the illness and identify any danger signs. Screening also included measuring respiratory rates using the rate timer, oxygen saturation using pulse oximetry with infant probes, and axillary temperature using a digital thermometer (**Table 1**). We classified the screened children by the HCP according to the IMCI protocol as: 1) cough or cold/no pneumonia, 2) pneumonia (fast breathing, respiratory rate 50 breaths per minute or more in children aged 2–11 months and 40 breaths or more in children aged 12–59 months or chest indrawing), or 3) severe pneumonia (any general danger sign or stridor in a calm child). Concurrently, the data collectors documented complete home addresses, contact numbers, and landmarks to reach out for day 15 follow-up for eligible enrolled children. They also recorded anthropometric measurements, including weight, height/length, and mid-upper arm circumference.

**Table 1:** Details of the implementation strategy of the study

Activity	What	Who	When	Where	How
Case identification	To identify all children 2–59 months presenting to the outpatient department with cough and/or difficult breathing	Healthcare provider	During the day one visit	Selected primary health care facilities	Clinical assessment for cough, difficult breathing, chest indrawing, and respiratory rate was conducted using the IMCI protocol
Screening and enrolment	To identify chest indrawing pneumonia in children 2–59 months	Screening and enrolment were done by IMCI-trained health care providers	During the day one visit	Selected primary health care facilities	A child identified with chest indrawing was enrolled in the study after consent was obtained
Treatment provision	As prescribed by IMCI protocols and relevant national guidelines and policy	Healthcare provider	During the day one visit	Selected primary health care facilities and at-home	Treatment with antibiotics. In the case of oral antibiotics, the mother/caretaker provides the antibiotic dose to the child. If the child was referred to a hospital, a pre-referral antibiotic dose was administered
Follow-up	To evaluate treatment outcome	Data collector	On day 15 after enrolment, the treatment outcome was documented	At the selected primary health care facilities, home or the hospital if the child was hospitalised	Data collectors conducted follow-up on day 15 visits and collected outcome data using application-based outcome documentation (A-1) on tablets
Treatment documentation	Prescription paper, medicine box, pictorial medicine chart or caretaker recall	Data collector	Day 15	At home/hospital	Data collectors conducted the day 15 visit and used an application-based outcome documentation form
Outcome assessment	As per the parent’s response, outcome status was reported (improved, same, worse, death)	Data collector	Day 15	At the home/hospital	Data collectors conducted the day 15 visit and used an application-based outcome documentation form

IMCI – Integrated Management of Childhood Illness

After the HCPs used the IMCI protocol to classify and treat children, we obtained written informed consent from parents/caregivers, and children were enrolled in the study. We collected information about demography, the child’s home address and the family’s telephone/mobile number on the study case report form. We made video clips of clinical signs, such as chest indrawing, for each enrolled child (without their face) after obtaining permission from parents/caregivers. We verified the parents’/caregivers' telephone/mobile numbers through confirmation calls, and authenticated addresses by mapping sketches of nearby landmarks to facilitate location identification.

We conducted validation of the screening process at the health facility level, while validation of follow-up visits and data collection was performed at the household level. At the health facility, 1.3% (n/N = 46/3526) randomly selected children presenting to the PHC with cough and difficult breathing were validated to assess the HCPs adherence to the IMCI protocol for assessment and classification, and for the use of the respiratory rate timer and pulse oximeter. When deficiencies were identified in assessment or classification by the HCPs when using the IMCI protocol, on-site refresher training was provided. A randomly selected sample of 7.5% (n/N = 34/452) of the children followed up in the study was validated to verify their health facility visits, the care provided by the HCP, the treatment received, and the data collector's visit to the household.

#### Follow-up and outcome assessment

Data collectors systematically followed up all enrolled children on day 15 post-enrolment within a permissible window of two days. We called the parents/caregivers of the child two to three days before the day 15 follow-up to confirm the child’s home address whenever needed. In cases where it was difficult to contact the parents/caregivers and reach the addresses provided, we contacted the local community health care workers, such as lady health workers, lady health visitors, and vaccinators from the expanded programme on immunisation, to reach the accurate household for the follow-up.

We recorded follow-up data collection electronically on handheld electronic devices. We captured detailed information on the child’s outcomes, whether the child recovered, remained the same, the condition worsened or the child died. Additionally, we documented the treatment provided, names of the antibiotics, route of administration, duration, frequency and adherence as well as details in case of hospitalisation. Parents’/caregivers’ confirmation corroborated the antibiotic use through the prescription provided by the HCP during the initial visit. If a prescription was unavailable, data collectors probed for evidence of used medication bottles or leaflets to identify the medication. In the absence of a prescription and medicine bottles, a pictorial chart of the commonly used antibiotics for pneumonia treatment in the community was shown to parents/caregivers to help identify the antibiotics administered to the child. Additionally, data on the parent’s education level was collected, specifically whether the mother, father or both parents were illiterate, could provide only a signature, had primary education (up to five years), had secondary education (up to 10 years), or had higher secondary education (up to 12 years). Data on the child’s immunisation status was based on either the vaccination card or on parents’/caregivers’ recall when the vaccination card was unavailable. We asked the respondent a series of probing questions regarding the child’s vaccination history, including inquiring about the route and site of vaccination administration and checking for a visible Bacillus Calmette-Guérin vaccination scar on the arm of the child. For children who had died, a verbal autopsy was conducted after the four to six weeks period following the child’s death to ascertain the possible cause of death. This involved using structured questionnaires and verbatim accounts to capture detailed information about the events leading to the child’s death [17].

### Ethical considerations

We obtained informed written consent from parents/caregivers before enrolment. The literate parents/guardians signed the informed consent form, while the illiterate parents/guardians put their thumbprint. Participation was voluntary, and declining to participate did not affect medical treatment. We obtained ethical approvals for this study from the Institutional Review Board of the People’s Primary Healthcare Initiative in Karachi, Sindh and from the WHO Ethical Review Committee before the commencement of the study. We strictly adhered to ethical guidelines for medical research involving human subjects.

### Registration

This research is part of a multi-country study evaluating current practices in the outpatient management of chest indrawing pneumonia within LMICs. The trial is registered at ISRCTN Registry with the number 12687253 [16].

### Sample size

Based on assumptions of a 5% case fatality ratio (CFR) for chest indrawing (taken from primary care data from Malawi; personal communication with Humphrey Nsona, May 2021), 3% margin of error, 95% confidence level and accounting for a 5% loss to follow-up, in the protocol, we calculated that the sample size for each study site was 310 children [16].

### Study outcomes

The primary outcome of our study was to estimate the CFR in children receiving outpatient treatment for chest indrawing pneumonia. The secondary outcome was to assess antibiotic use, hospital admissions and treatment adherence among these children.

### Data analysis

We inspected data for completeness. We performed data analysis using STATA, version 17.0 (Stata Corp LLC, Texas, USA). We summarised baseline characteristics of children aged 2–59 months with chest indrawing pneumonia as frequencies with percentages for categorical data and means (x̄), standard deviations (SDs) and medians with inter-quartile ranges for continuous variables. We calculated the primary outcome CFR as the number of deaths on day 15 post-enrolment divided by the total number of enrolled children who were followed up in the study and was reported with a 95% confidence interval (CI). In addition, we calculated the incidence rates with 95% CI per 1000 child-days for death and hospitalisation. For anthropometric measures, we calculated Z-scores based on the WHO 2006 growth reference standards [18]. For immunisation, we classified children as fully immunised if they had received all age-appropriate vaccinations according to the expanded programme of immunisation schedule in Pakistan.

Two physicians independently reviewed the collected verbal autopsies data to determine the cause of death. In case of discordance, a third expert physician was available for conflict resolution and to determine the probable cause of death.

## RESULTS

**Figure 2:**
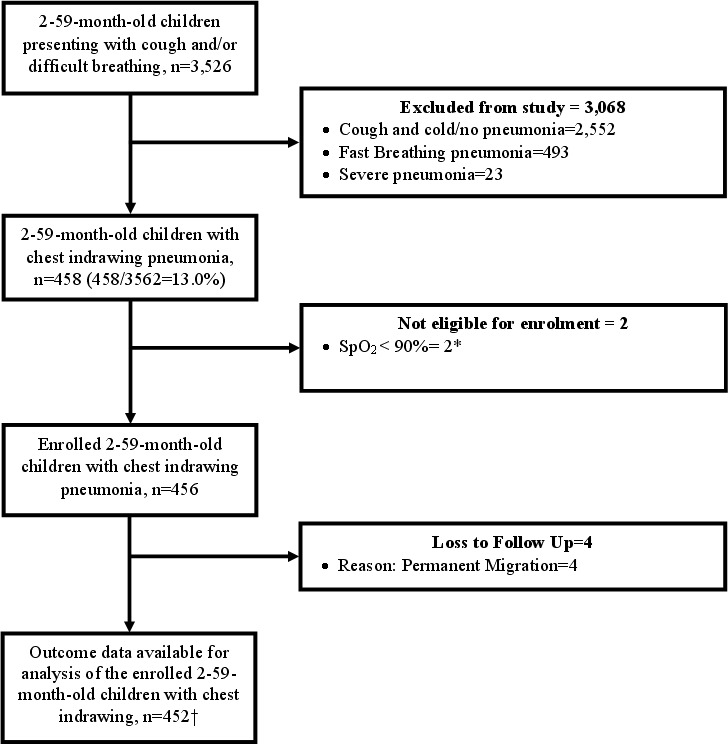
Flowchart of the study. *Two children had hypoxemia (SpO2 < 90 at enrolment) and were referred to a referral facility. Both were admitted and received inpatient treatment, one child was discharged after five days and the other one was discharged on the third day of hospitalisation. †Two children died.

From December 2022 to March 2024, we screened 3526 children aged 2–59 months presenting with cough and/or difficult breathing. Of these, 458 children had chest indrawing pneumonia. Two were excluded due to SpO_2 _< 90 and were immediately referred to a higher facility for further management. We enrolled 456 children, four of whom were lost to follow-up due to permanent migration, thus, on day 15 we followed up 452 children (**Figure 2**).

### Baseline demographic and clinical characteristics

Among 456 enrolled children, 275 (60.3%) were male, 297 (65.1%) were aged 2–11 months, and 150 (32.9%) had a temperature of 38°C or more. The respiratory rate was x̄ = 58.0 breaths per minute (SD = 6.47) for children aged 2–11 months and x̄ = 54.5 (SD = 7.79) for children aged 12–59 months. 119 (26.1%) had height for age Z-score<–3 SD (stunted), 70 (15.4%) had weight for height Z-score<–3 SD (severe acute malnutrition) and 139 (30.5%) had weight for age Z-score<–3 SD. Both parents were uneducated (no schooling) for 332 (72.8%) children. At follow-up on day 15, 437 (95.6%) of primary respondents were mothers. Only 70 (15.5%) children were fully immunised (**Table 2**; Table S1 in the **Online Supplementary Document**). There were no significant associations between sociodemographic factors, immunisation status, and treatment outcomes (Table S2 in the **Online Supplementary Document**).

**Table 2:** Baseline demographic and clinical characteristics of children aged 2–59 months with chest indrawing pneumonia (n = 456)*

Characteristics	Children aged 2–59 months with chest indrawing pneumonia
Name of the participating health facility	
*BHU plus, Dhabeji*	131 (28.7)
*RHC Varr*	156 (34.2)
*RHC, Jungshahi*	169 (37.1)
Age in months	
*x̄ (SD)*	12.8 (12.6)
*MD (IQR)*	7.0 (4.0–17.0)
Age category in months	
*2–11*	297 (65.1)
*12–59*	159 (34.9)
Sex	
*Male*	275 (60.3)
*Female*	181 (39.7)
Axillary temperature in °C	
*x̄ (SD)*	37.9 (0.7)
*MD (IQR)*	37.8 (37.7–38.3)
Axillary temperature categories in °C	
*<37.0*	47 (10.3)
*37.0–37.9*	217 (47.6)
*≥38.0–38.9*	150 (32.9)
*Missing values†*	42 (9.2)
Parent’s education	
*Both uneducated*	332 (72.8)
*Both educated*	36 (7.9)
*Only father educated*	70 (15.4)
*Only mother educated*	13 (2.9)
*Missing‡*	5 (1.1)
Respiratory rate per minute (age 2–11 months)	
*x̄ (SD)*	58.03 (6.47)
*MD (IQR)*	57.0 (53.0–60.0)
Respiratory rate per minute (age 12–59 months)	
*x̄ (SD)*	54.50 (7.79)
*MD (IQR)*	54.0 (48.0–59.0)
SpO_2_	
*x̄ (SD)*	95.9 (2.6)
*MD (IQR)*	96.0 (94.0–98.0)
SpO_2_ categories	
*90–93*	29 (6.4)
*94–100*	121 (26.5)
*Missing values§*	306 (67.1)
Adult primary respondent at the time of follow up	
*Mother*	437 (95.8)
*Father*	3 (0.7)
*Grandfather*	1 (0.2)
*Grandmother*	9 (2.0)
*Other*	2 (0.4)
*Missing^¶^*	4 (0.9)
WAZ	
*<–3*	139 (30.5)
*–3≤WAZ<–2*	109 (23.9)
*≥–2*	208 (45.6)
HAZ	
*<–3*	119 (26.1)
*–3≤HAZ<–2*	90 (19.7)
*≥–2*	247 (54.2)
WHZ	
*<–3*	70 (15.4)
*–3≤WHZ<–2*	81 (17.8)
*≥–2*	305 (66.9)
MUAC in cm (n = 287)‖	
*≤11.5*	160 (55.7)
*11.5–12.5*	94 (32.7)
*≥12.5*	33 (11.5)
Immunisation status**	
*Fully immunised*	70 (15.4)
*Partially immunised*	305 (66.9)
*Not immunised*	77 (16.9)
*Missing values*	4 (0.9)

BHU – basic health unit, HAZ – height for age Z-score, IQR – interquartile range, MD – median, MUAC – mid-upper arm circumference, RHC – rural health centre, SD – standard deviation, SpO_2_ – peripheral oxygen saturation, WAZ – weight for age Z-score, WHZ – Weight for height Z-score, x̄ – mean

*Presented as n (%) unless specified otherwise.

†We did not collect information regarding temperature measurements during the first month of study.

‡Parents’ education information is missing for five children. Four were lost to follow-up and one died.

§Pulse oximeter measurements were performed between October 2023 and March 2024, when pulse oximeters became available at the health facilities.

¶Children lost to follow-up.

‖MUAC was measured only in children aged ≥6 months.

**Data on immunisation status was available only for 452 children.

### Primary outcome

Two deaths were reported among the 452 followed-up children, giving a CFR of 0.4% (95% CI = 0.05–1.59). Both deaths were in children aged 2–11 months (CFR = 0.7; 95% CI = 0.08–2.56). The overall incidence rate of death was 0.3 (95% CI = 0.03–1.07) per 1000 child-days, while it was 0.5 (95% CI = 0.001–1.66) per 1000 child-days among children aged 2–11 months (**Box 1**).

Box 1 **Verbal autopsy details of deaths.**

**Case 1:** A four-month-old male child presented at a primary health care facility with a two-day history of fever, cough, and difficulty in breathing. At enrolment, he was classified with chest indrawing pneumonia and prescribed oral cefixime treatment for five days at home. His respiratory rate was 58 breaths per minute, weight was 4 kg, length was 56 cm, weight for height Z-score was –2.28 and he was partially immunised, having received only two doses of oral polio vaccine. However, his condition did not improve, and his parents sought medical consultation at a private clinic, where he was given outpatient treatment and advised hospitalisation. However, the family declined the medical advice for inpatient care and instead visited multiple private local clinics and received different treatments, none improving the child’s condition, which deteriorated over time. By day 14, the child’s condition further deteriorated and he developed seizures along with high-grade fever, cough, and severe difficulty in breathing, following which he was immediately taken to the nearest primary health care facility, where he was given some injections (contents unknown) and was yet again advised to seek immediate hospital admission, but the family did not follow the medical advice, and the child eventually died at home on the day 14 of his illness. Based on verbal autopsy information reviewed by two independent physicians, the possible cause was assigned as either meningitis or septicaemia.

**Case 2:** An eight-month-old male child, presented to the primary health facility with a history of cough and difficult breathing, was classified as having chest indrawing pneumonia and prescribed oral amoxicillin for five days at home. His respiratory rate was 52 breaths per minute, weight was 6.9 kg, length was 68 cm, weight for height Z-score was –1.28 and he was partially immunised with Bacillus Calmette-Guérin, oral polio vaccine 1 and 2 (OPV-1 & 2), pneumococcal conjugate vaccine 2 (PCV-2) and Rotavirus-2 (ROTA-2) vaccines. According to the mother, the child was given antibiotics as advised, his condition improved and was declared cured by the physician at a follow-up visit. As per the mother’s statement on the day 14 of enrolment, the child was active, alert and playful throughout the day. During the late hours of the night, the mother breastfed her infant under conditions of limited visibility due to a power outage, accompanied by severe cold weather. Following the feeding, she covered the infant with two blankets to ensure warmth. In the early morning hours, when the mother attempted to wake the infant for another feeding, she discovered that the child was unresponsive, with milk present in the mouth and nostrils; the infant had died. A verbal autopsy, reviewed by two independent physicians, suggested that the most likely cause of death was either aspiration or suffocation.

### Secondary outcomes

Among the 450 children who survived, the incidence of hospitalisation was 0.3 (95% CI = 0.03–1.07) per 1000 child days. A 12-month-old male child was admitted to a private hospital four days after enrolment. Oxygen saturation was not assessed at enrolment. He was hospitalised for four days and was first treated with injected ampicillin four times daily for three days. Treatment was subsequently changed to an injection of ceftriaxone once daily for three days plus zinc syrup twice daily for three days. A four-month-old male child was admitted to a government hospital one day after enrolment. His SpO_2_ was 96% at enrolment. He was hospitalised for two days and was first treated with injectable ampicillin thrice daily for three days, followed by an injection of ceftriaxone twice daily for three days plus injectable hydrocortisone twice daily for three days. The duration from enrolment to hospitalisation for the two cases was x̄ = 2.5 days (SD = 2.12). Both children survived. When stratified by age category, the incidence of hospitalisation was 0.2 (95% CI = 0.001–1.28) per 1000 child-days for children aged 2–11 months and 0.4 (95% CI = 0.01–2.33) per 1000 child-days for children aged 12–59 months.

All enrolled children received oral antibiotics for outpatient management of chest indrawing pneumonia prescribed by HCPs. Among 452 children, 282 (62.4%) received oral amoxicillin, while 114 (25.2%) were prescribed cefixime, primarily at the rural health centre Jungshahi (Table S4 in the **Online Supplementary Document**). Oral amoxicillin for five days or more was received by 236 (N = 282, 83.7%). A higher adherence rate of 88.0% was observed among children aged 12–59 months. Collectively in both age groups, 46 (16.3%) children were non-adherent to five days of therapy (**Table 3**).

**Table 3:** Details of antibiotics taken by the age of the child followed up after 15 days*

Items	Follow-up children	Children aged 2–11 months	Children aged 12–59 months
Oral antibiotics given to followed up children	n = 452	n = 293	n = 159
*Amoxicillin*	282 (62.4)	207 (70.6)	75 (47.2)
*Cefixime*	114 (25.2)	62 (21.2)	52 (32.7)
*Amoxicillin/clavulanic acid*	25 (5.5)	10 (3.4)	15 (9.4)
*Azithromycin*	18 (4.0)	9 (3.1)	9 (5.7)
*Other antibiotics^†^*	13 (2.9)	5 (1.7)	8 (5.0)
Amoxicillin duration in days‡	n = 282	n = 207	n = 75
*<5*	46 (16.3)	37 (17.9)	9 (12.0)
*5*	154 (54.6)	112 (54.1)	42 (56.0)
*6–8*	81 (28.7)	57 (27.5)	24 (32.0)
*Missing§*	1 (0.4)	1 (0.5)	0 (0.0)

*Presented as n (%).

†Other medicines including Cefpodoxime, Cephalexin, Ciprofloxacin, Sulfamethoxazole-trimethoprim, Fosfomycin, and Linezolid.

‡The IMCI protocol recommends oral amoxicillin twice daily for five days.

§One child who died does not have information on the amoxicillin dose.

## DISCUSSION

In this prospective cohort study from Pakistan, we observed a very low CFR (0.4%) among enrolled children, and only one death was related to the pneumonia episode. All enrolled children who received oral antibiotics, oral amoxicillin was prescribed and taken by nearly two-thirds of the patients, and over 80% were adherent to the treatment regimen. The majority of children were cured, and only two children were hospitalised during treatment. One death and two hospitalisations were treated with oral cefixime and not oral amoxicillin. Four cases were lost to follow-up. Only 15% of enrolled children were fully immunised.

In the current study, we observed <1% CFR among children aged 2–59 months with chest indrawing pneumonia. Only two deaths were reported in our study, which is similar to data reported from three observational studies that treated chest indrawing pneumonia with oral amoxicillin in children aged 2–59 months. A community-based study conducted in Kenya [19] reported a CFR of 0.26%, and no deaths were reported from an outpatient-based study from Papua New Guinea [20] nor from a study from four countries named Bangladesh, Egypt, Ghana and Vietnam [21]. Three multi-site community-based randomised controlled trials, two conducted in Pakistan and one in four countries named Bangladesh, Ethiopia, India and Malawi, reported <1% CFR where community-level health workers (CLHW) treated children aged 2–59 months with chest indrawing pneumonia with oral amoxicillin at home [22–24]. The above-mentioned trials show that the management of chest indrawing pneumonia in children aged 2–59 months without any danger signs or hypoxaemia with oral amoxicillin at home is successful and safe. This is also supported by a systematic review [7]. However, other studies have reported higher CFRs, which can be attributed to variations in the study population, follow-up duration, and the period during which the studies were conducted. A study conducted in semi-urban areas near Karachi, Sindh, utilising household surveillance for 16 months, reported CFRs of 1.4% for fast breathing and 1.9% for chest indrawing pneumonia, which might reflect limited access to health care services and the inclusion of cases where children might not have sought appropriate health care [25]. Another study based on hospital records from four years (1989–1992) reported CFRs ranging from 2.3–7.8% for fast breathing and chest indrawing respectively, reflecting the management protocols and treatment options available during that time [26].

The child who died due to pneumonia complications did not receive appropriate care because parents refused referral advice for inpatient care. Delays in appropriate care, including refusal to accept referral advice for inpatient care, are associated with an increased risk of death due to pneumonia [27]. Unfortunately, refusal to accept referral advice is fairly common in many LMICs because of various factors like finances, travel costs, distance to health care facilities, cultural norms, past negative experiences with quality of care and the attitude of health workers at the hospital [28,29].

In our study, the health workers’ adherence to the IMCI protocol regarding assessment and classification was high. All patients were treated with oral antibiotics, and none were referred for hospitalisation except for the two cases with hypoxaemia. Only two-thirds of the patients were advised oral amoxicillin, of which over 80% were cured. However, not all children with severe malnutrition were referred to a hospital per IMCI recommendation. Reports of non-adherence to various components of the IMCI chart booklet have been reported from several countries [30–33]. Sometimes, this non-adherence to IMCI guidance is because of a lack of knowledge or non-availability of appropriate antibiotics, but it can also be due to a lack of motivation to adhere to the IMCI protocol, sometimes due to poor remuneration [34]. We had a very low rate of loss to follow-up, <1%, which was much less than that reported by other observational chest indrawing pneumonia management studies [19–21]. This low loss to follow-up can be attributed to a stable community with no frequent migration, call reminders before follow-up visits, thorough mapping of households and collaboration with CLHWs to reach households of those enrolled children whose families were not reachable by telephone. In our study, only 15% of children were fully vaccinated, which is worrisome. This calls for public health interventions to promote the uptake of routine immunisation, particularly for measles, *Haemophilus influenzae* type b and *Pneumococcal* conjugate vaccines to reduce the incidence of pneumonia and associated mortality in children under five years of age.

The implementation of outpatient management for chest indrawing pneumonia with oral amoxicillin can reduce health care costs, particularly in LMICs, including Pakistan, where health systems are overburdened, and where families face financial challenges to seek care at referral facilities. Hussain et al. [35] reported that the average cost for outpatient management of childhood pneumonia was USD 13.4. In comparison, management in a hospital in the northern areas of Pakistan costs USD 71.0. In further economic analyses of childhood pneumonia treatment, the total societal cost for treatment of a pneumonia episode was estimated to be USD 22.6 compared to USD 142.9 for severe pneumonia [12]. Moreover, medicines (40.5%) comprised the highest proportion of household expenditure, followed by meals (23.7%), hospitalisation (13.2%) and transport costs (12.2%) [12]. In another community-based study in Pakistan [36], the average household cost of treatment of pneumonia by a CLHW was USD 1.5 compared with USD 7.6 for those referred to a hospital. An analysis of 74 LMICs found the 2012 WHO pneumonia management guidelines [8] were more cost-effective compared to the 2005 WHO guidelines [11,37]. For Pakistan, the hospital costs were estimated to be one-sixth using the 2012 protocol compared to that of 2005. This reduction was primarily due to the management of chest indrawing pneumonia at home with oral amoxicillin instead of hospitalisation [11]. Therefore, the pneumonia guideline of treating chest indrawing pneumonia at home with oral amoxicillin saves money for the health system and is also favourable to families, substantially reducing costs for medicines, meals, transport and other related issues [38].

All children in the current study were treated at home with oral antibiotics. Health-care-associated infections (HAIs) are more common in LMICs compared to high-income countries and lead to substantial morbidity and mortality [39,40]. These infections often result in severe complications, prolonged hospital stays, and a risk of contracting additional severe infections, thus complicating the initial disease and requiring extreme medical interventions increasing health care costs [41] A study analysing point prevalence surveys from 99 countries reported that 136 million episodes of HAI occurred yearly, with the highest burden in China, India and Pakistan [42]. Outpatient management of pneumonia can considerably reduce the burden of HAI and additional associated costs borne by the health system and families, particularly in low-resource settings, allowing more efficient utilisation of limited resources.

The IMCI strategy provides protocols for standard case management of common childhood illnesses, including pneumonia. Standard case management saves lives and reduces unnecessary antibiotic use [26,43–45]. Inappropriate and unnecessary antibiotic use increases antimicrobial resistance (AMR), which is now a global public health emergency [46]. An estimated 7.7 million deaths are attributed to bacterial infections globally, of which 1.3 million deaths are caused by resistant bacterial pathogens [47]. AMR is a critical health issue in Pakistan, with an increased emergence of multidrug-resistant pathogens [48]. The application of evidence-based standard case management for the outpatient treatment of chest indrawing pneumonia with oral amoxicillin will reduce the misuse and overuse of antibiotics, not only benefiting individual patient outcomes but also safeguarding public health by reducing the spread of resistant bacteria. Promoting rational antibiotic use will contribute to reducing AMR pressure and mitigating drug-resistant pneumonia in children. Our study illustrates the benefits of standard case management using the IMCI protocol at PHC facilities and at the outpatient departments of small hospitals.

This study has several strengths. Integration of this study into the primary health care delivery system in Thatta district ensured real-time identification of pneumonia cases, reflecting the actual burden and care-seeking practices in the community. We were able to enrol a substantial number of pneumonia cases with no refusals to participate, which enhanced the reliability of our findings. The negligible loss to follow-up reduced the potential for attrition bias, thus ensuring the representativeness of the results. The use of a standardised case management IMCI protocol by the HCPs for the assessment, classification, and management of pneumonia was quite encouraging. These results could be reflective of similar socio-economic conditions and settings.

Our study had some limitations. The primary outcome status was reported as per the respondent's statements on day 15 post-enrolment, which could introduce a misclassification bias. To mitigate this, data collectors used probing questions to elicit detailed information about the child’s condition to verify the parent’s statements. Despite these measures, the reliance on parental reports could still introduce some level of bias, particularly recall bias, in cases where parents/caregivers may inaccurately remember details about their child’s illness or the treatment administered, affecting the accuracy of the collected data. Another challenge was the intermittent stockouts of oral amoxicillin at the primary health facilities during the study. This may have influenced the local prescribing practices at these facilities. Such logistical challenges are commonly encountered in resource-limited settings contributing to the observed treatment patterns. We found that 54.4% of caregivers obtained antibiotics free of cost from health care facilities, while 45.1% had to purchase them from external vendors and 0.41% did not purchase the antibiotic (Table S3 in the **Online Supplementary Document**). This indicates potential stockouts at PHC-level facilities. However, since oral amoxicillin was inexpensive and widely available from vendors, this workaround did not negatively impact adherence.

## CONCLUSIONS

Our findings support the use of the IMCI protocol to treat chest indrawing pneumonia in children aged 2–59 months with a five-day course of oral amoxicillin on an outpatient basis. This intervention has the potential to reduce childhood pneumonia deaths, increase access to treatment, improve treatment coverage, reduce referrals and pressure on the overburdened referral health facilities and reduce costs for the health system and families in resource-limited settings.

## Additional material


Online Supplementary Document

## References

[1] The United Nations International Children’s Emergency Fund. Under-five child mortality. 2024. Available: https://data.unicef.org/topic/child-survival/under-five-mortality/. Accessed: 22 April 2024.

[2] The United Nations International Children’s Emergency FundPneumonia. 2024. Available: https://data.unicef.org/topic/child-health/pneumonia/. Accessed: 21 June 2024.

[3] The United Nations International Children’s Emergency Fund. UNICEF Data: Pakistan. 2019. Available: https://data.unicef.org/country/pak/. Accessed: 24 July 2024.

[4] United Nations Inter-agency Group for Child Mortality Estimation. Child Mortality Estimates. 2024. Available: https://childmortality.org/. Accessed: 13 January 2025.

[5] The United Nations International Children’s Emergency Fund. One child dies of pneumonia every 39 seconds, agencies warn. Pneumonia – a preventable disease – kills more children than any other infection. 2019. Available: https://www.unicef.org/pakistan/press-releases/one-child-dies-pneumonia-every-39-seconds-agencies-warn. Accessed: 24 July 2024.

[6] United Nations Inter-agency Group for Child Mortality Estimation. Levels and Trends in Child Mortality: Report 2022. New York, New York, USA: United Nations. 2023. Available: https://data.unicef.org/resources/levels-and-trends-in-child-mortality-2024/#:~:text=This%20year's%20United%20Nations%20Inter,%E2%80%935.4)%20million%20in%202022. Accessed: 25 July 2024.

[7] Wilkes C Graham H Walker P Duke T ARI Review group Which children with chest indrawing pneumonia can be safely treated at home, and under what conditions is it safe to do so? A systematic review of evidence from low- and middle-income countries. J Glob Health. 2022;12:10008. 36040992 10.7189/jogh.12.10008 PMC9428503

[8] World Health Organization. Recommendations for the management of common childhood conditions. Geneva, Switzerland: World Health Organization; 2012. Available: https://apps.who.int/iris/bitstream/handle/10665/44774/9789241502825_eng.pdf. Accessed: 29 April 2024.

[9] World Health Organization. Revised WHO classification and treatment of childhood pneumonia at health facilities. 2014. Available: https://www.who.int/publications/i/item/9789241507813. Accessed: 29 April 2024.

[10] World Health Organization. Integrated Management of Childhood Illness (IMCI): Chart Booklet. 2014. Available: https://www.who.int/teams/maternal-newborn-child-adolescent-health-and-ageing/child-health/integrated-management-of-childhood-illness. Accessed: 29 August 2024.

[11] Zhang S Incardona B Qazi SA Stenberg K Campbell H Nair H Cost-effectiveness analysis of revised WHO guidelines for management of childhood pneumonia in 74 Countdown countries. J Glob Health. 2017;7:010409. 10.7189/jogh.07.010409 28400955 PMC5344007

[12] Hussain H Waters H Khan AJ Omer SB Halsey NA Economic analysis of childhood pneumonia in Northern Pakistan. Health Policy Plan. 2008;23:438–42. 10.1093/heapol/czn033 18755733

[13] World Health Organization. Integrated Management of Childhood Illness: Chartbooklet Pakistan. Geneva, Switzerland: World Health Organization; 2019. Available: https://sites.pitt.edu/~super1/Pirzado/Chartbooklet%202019%20Pakistan.pdf. Accessed: 25 July 2024.

[14] Pakistan Federal Bureau of Statistics. Census 2017. 2017. Available: https://www.pbs.gov.pk/. Accessed: 2 May 2024.

[15] United Nations Office for the Coordination of Humanitarian Affairs. Common Operational Datasets – Administrative Boundaries Pakistan. 2024. Available: https://data.humdata.org/dataset/cod-ab-pak. Accessed: 13 March 2025.

[16] Cipam Study Group CIPM Understanding the outcome and management of children aged 2-59 months with chest indrawing pneumonia: a study protocol for an observational study in Ethiopia, India, Nigeria, Pakistan, Uganda and Zambia. BMJ Open. 2024;14:e084350. 10.1136/bmjopen-2024-084350 38904143 PMC11191814

[17] Nichols EK Byass P Chandramohan D Clark SJ Flaxman AD Jakob R WHO Verbal Autopsy Working Group The WHO 2016 verbal autopsy instrument: An international standard suitable for automated analysis by InterVA, InSilicoVA, and Tariff 2.0. PLoS Med. 2018;15:e1002486. 10.1371/journal.pmed.1002486 29320495 PMC5761828

[18] World Health Organization. WHO child growth standards. 2006. Available: https://www.who.int/publications/i/item/924154693X. Accessed: 29 April 2024.

[19] Onono M Abdi M Mutai K Asadhi E Nyamai R Okoth P Community case management of lower chest indrawing pneumonia with oral amoxicillin in children in Kenya. Acta Paediatr. 2018;107:44–52. 10.1111/apa.14405 30570795

[20] Morre R Sobi K Pameh W Ripa P Vince JD Duke T Safety, Effectiveness and Feasibility of Outpatient Management of Children with Pneumonia with Chest Indrawing at Port Moresby General Hospital, Papua New Guinea. J Trop Pediatr. 2019;65:71–7. 10.1093/tropej/fmy013 29660106 PMC6366396

[21] Addo-Yobo E Anh DD El-Sayed HF Fox LM Fox MP MacLeod W Outpatient treatment of children with severe pneumonia with oral amoxicillin in four countries: the MASS study. Trop Med Int Health. 2011;16:995–1006. 10.1111/j.1365-3156.2011.02787.x 21545381 PMC3154370

[22] Bari A Sadruddin S Khan A Khan I Khan A Lehri IA Community case management of severe pneumonia with oral amoxicillin in children aged 2-59 months in Haripur district, Pakistan: a cluster randomised trial. Lancet. 2011;378:1796–803. 10.1016/S0140-6736(11)61140-9 22078721 PMC3685294

[23] Soofi S Ahmed S Fox MP MacLeod WB Thea DM Qazi SA Effectiveness of community case management of severe pneumonia with oral amoxicillin in children aged 2–59 months in Matiari district, rural Pakistan: a cluster-randomised controlled trial. Lancet. 2012;379:729–37. 10.1016/S0140-6736(11)61714-5 22285055

[24] EMPIC Study Group Innovative, enhanced community management of non-hypoxaemic chest indrawing pneumonia in 2-59-month-old children: a cluster-randomised trial in Africa and Asia. BMJ Glob Health. 2022;7:e006405. 10.1136/bmjgh-2021-006405 34987033 PMC8734014

[25] Owais A Tikmani SS Sultana S Zaman U Ahmed I Allana S Incidence of pneumonia, bacteremia, and invasive pneumococcal disease in Pakistani children. Trop Med Int Health. 2010;15:1029–36. 10.1111/j.1365-3156.2010.02591.x 20636300

[26] Qazi SA Rehman GN Khan MA Standard management of acute respiratory infections in a children’s hospital in Pakistan: impact on antibiotic use and case fatality. Bull World Health Organ. 1996;74:501–7. 9002330 PMC2486861

[27] The United Nations International Children’s Emergency Fund. Pneumonia: the forgotten killer of children. 2006. Available: https://www.who.int/publications/i/item/9789280640489. Accessed: 13 March 2025.

[28] Kozuki N Guenther T Vaz L Moran A Soofi SB Kayemba CN A systematic review of community-to-facility neonatal referral completion rates in Africa and Asia. BMC Public Health. 2015;15:989. 10.1186/s12889-015-2330-0 26419934 PMC4589085

[29] Applegate JA Ahmed S Harrison M Callaghan-Koru J Mousumi M Begum N Caregiver acceptability of the guidelines for managing young infants with possible serious bacterial infections (PSBI) in primary care facilities in rural Bangladesh. PLoS One. 2020;15:e0231490. 10.1371/journal.pone.0231490 32287286 PMC7156040

[30] Krüger C Heinzel-Gutenbrunner M Ali M Adherence to the integrated management of childhood illness guidelines in Namibia, Kenya, Tanzania and Uganda: evidence from the national service provision assessment surveys. BMC Health Serv Res. 2017;17:822. 10.1186/s12913-017-2781-3 29237494 PMC5729502

[31] Kpoda HBN Some SA Somda MAS Yara M Dabone BEA Ilboudo P Evaluation of Adherence of Health-Care Workers to Integrated Management of Childhood Illness Guidelines in the Context of the Free Care Program in Burkina Faso. Am J Trop Med Hyg. 2022;107:610–6. 35895336 10.4269/ajtmh.21-0976 PMC9490671

[32] Senn N Rarau P Salib M Manong D Siba P Rogerson S Use of antibiotics within the IMCI guidelines in outpatient settings in Papua New Guinean children: an observational and effectiveness study. PLoS One. 2014;9:e90990. 10.1371/journal.pone.0090990 24626194 PMC3953204

[33] El-Ayady AA Meleis DE Ahmed MM Ismaiel RS Primary Health Care Physicians’ Adherence and Attitude Towards Integrated Management of Childhood Illness Guidelines in Alexandria Governorate in Egypt. Glob J Health Sci. 2015;8:217–24. 10.5539/gjhs.v8n5p217 26652094 PMC4877194

[34] Lange S Mwisongo A Mæstad O Why don’t clinicians adhere more consistently to guidelines for the Integrated Management of Childhood Illness (IMCI)? Soc Sci Med. 2014;104:56–63. 10.1016/j.socscimed.2013.12.020 24581062

[35] Hussain H Waters H Omer SB Khan A Baig IY Mistry R The cost of treatment for child pneumonias and meningitis in the Northern Areas of Pakistan. Int J Health Plann Manage. 2006;21:229–38. 10.1002/hpm.847 17044548

[36] Sadruddin S Shehzad S Bari A Khan A Ibad Ul H Khan A Household costs for treatment of severe pneumonia in Pakistan. Am J Trop Med Hyg. 2012;87:137–43. 10.4269/ajtmh.2012.12-0242 23136289 PMC3748514

[37] World Health Organization. Pocket book of hospital care for children: guidelines for the management of common illnesses with limited resources. Geneva, Switzerland: World Health Organization; 2005. Available: https://www.afro.who.int/sites/default/files/2017-06/pocket_booklet_hospital_care.pdf. Accessed: 5 September 2024.

[38] Patel AB Bang A Singh M Dhande L Chelliah LR Malik A A randomized controlled trial of hospital versus home based therapy with oral amoxicillin for severe pneumonia in children aged 3 - 59 months: The IndiaCLEN Severe Pneumonia Oral Therapy (ISPOT) Study. BMC Pediatr. 2015;15:186. 10.1186/s12887-015-0510-9 26577943 PMC4650851

[39] Allegranzi B Bagheri Nejad S Combescure C Graafmans W Attar H Donaldson L Burden of endemic health-care-associated infection in developing countries: systematic review and meta-analysis. Lancet. 2011;377:228–41. 10.1016/S0140-6736(10)61458-4 21146207

[40] Rosenthal VD Al-Abdely HM El-Kholy AA Alkhawaja SAA Leblebicioglu H Mehta Y International Nosocomial Infection Control Consortium report, data summary of 50 countries for 2010-2015: device-associated module. Am J Infect Control. 2016;44:1495–504. 10.1016/j.ajic.2016.08.007 27742143

[41] Gidey K Gidey MT Hailu BY Gebreamlak ZB Niriayo YL Clinical and economic burden of healthcare-associated infections: A prospective cohort study. PLoS One. 2023;18:e0282141. 10.1371/journal.pone.0282141 36821590 PMC9949640

[42] Balasubramanian R Van Boeckel TP Carmeli Y Cosgrove S Laxminarayan R Global incidence in hospital-associated infections resistant to antibiotics: An analysis of point prevalence surveys from 99 countries. PLoS Med. 2023;20:e1004178. 10.1371/journal.pmed.1004178 37310933 PMC10263350

[43] Bhandari N Mazumder S Taneja S Sommerfelt H Strand TA IMNCI Evaluation Study Group Effect of implementation of Integrated Management of Neonatal and Childhood Illness (IMNCI) programme on neonatal and infant mortality: cluster randomised controlled trial. BMJ. 2012;344:e1634. 10.1136/bmj.e1634 22438367 PMC3309879

[44] Rakha MA Abdelmoneim AN Farhoud S Pièche S Cousens S Daelmans B Does implementation of the IMCI strategy have an impact on child mortality? A retrospective analysis of routine data from Egypt. BMJ Open. 2013;3:e001852. 10.1136/bmjopen-2012-001852 23355663 PMC3563136

[45] Gouws E Bryce J Habicht JP Amaral J Pariyo G Schellenberg JA Improving antimicrobial use among health workers in first-level facilities: results from the multi-country evaluation of the Integrated Management of Childhood Illness strategy. Bull World Health Organ. 2004;82:509–15. 15508195 PMC2622903

[46] Antimicrobial Resistance Collaborators Global burden of bacterial antimicrobial resistance in 2019: a systematic analysis. Lancet. 2022;399:629–55. 10.1016/S0140-6736(21)02724-0 35065702 PMC8841637

[47] Okeke IN de Kraker ME Van Boeckel TP Kumar CK Schmitt H Gales AC The scope of the antimicrobial resistance challenge. Lancet. 2024;403:2426–38. 10.1016/S0140-6736(24)00876-6 38797176

[48] World Health Organization. Global antimicrobial resistance surveillance system (GLASS) report: early implementation 2017-2018. Geneva, Switzerland: World Health Organization; 2018. Available: https://iris.who.int/bitstream/handle/10665/277175/WHO-WSI-AMR-2018.4-eng.pdf. Accessed: 13 March 2025.

