# Supplementary material for: Outcome and management of children with chest indrawing pneumonia at primary health care settings in Pakistan: an observational cohort study
Source: J Glob Health. 2025 Mar 28;15:04096. doi: 10.7189/jogh.15.04096 (PMC11952181; doi:10.7189/jogh.15.04096)
Supplement: Online Supplementary Document [file jogh-15-04096-s001.pdf]

Supplement to: Suhag ZH, Pal A, Naeem M, Ahmed I, Khuwaja NA, Khakwani S, Mujtaba A, Qazi S, Nisar YB. Outcome and management of children with chest indrawing pneumonia at primary healthcare settings in Pakistan: an observational cohort study. J Glob Health. 2025;15:04096.

Table S1: Routine Immunisation coverage (Antigen wise as per EPI programme)

	TOTAL=452
**Immunisation Status**	**N = 452**
Fully immunized[¥]	70 (15.5)
Partially immunized	305 (67.5)
Not immunized	77 (17.0)
**At Birth**	**N = 452**
**Bacillus Calmette-Guérin (BCG)**	
Antigen Not Received	90 (19.9)
Antigen Received (Based on Card or Recall)	362 (80.1)
**Oral Polio Vaccine (OPV0)**	
Antigen Not Received	82 (18.1)
Antigen Received (Based on Card or Recall)	370 (81.9)
**At 6 Weeks**	**N = 452**
**Pentavalent (Penta1)**	
Antigen Not Received	132 (29.2)
Antigen Received (Based on Card or Recall)	320 (70.8)
**Oral Polio Vaccine (OPV1)**	
Antigen Not Received	272 (60.2)
Antigen Received (Based on Card or Recall)	180 (39.8)
**Pneumococcal Conjugate Vaccine (PCV1)**	
Antigen Not Received	138 (30.5)
Antigen Received (Based on Card or Recall)	314 (69.5)
**Rotavirus vaccine (RV1)**	
Antigen Not Received	139 (30.8)
Antigen Received (Based on Card or Recall)	313 (69.2)
**At 10 Weeks**	**N = 423**
**Pentavalent (Penta2)**	
Antigen Not Received	178 (42.1)
Antigen Received (Based on Card or Recall)	245 (57.9)
**Oral Polio Vaccine (OPV2)**	
Antigen Not Received	298 (70.4)
Antigen Received (Based on Card or Recall)	125 (29.6)
**Pneumococcal Conjugate Vaccine (PCV2)**	
Antigen Not Received	180 (42.6)
Antigen Received (Based on Card or Recall)	243 (57.4)
**Rotavirus vaccine (RV2)**	
Antigen Not Received	177 (41.8)
Antigen Received (Based on Card or Recall)	246 (58.2)
**At 14 Weeks**	**N = 381**
**Pentavalent (Penta3)**	
Antigen Not Received	212 (55.6)
Antigen Received (Based on Card or Recall)	169 (44.4)

**Oral Polio Vaccine (OPV3)**	
Antigen Not Received	306 (80.3)
Antigen Received (Based on Card or Recall)	75 (19.7)
**Pneumococcal Conjugate Vaccine (PCV3)**	
Antigen Not Received	217 (57.0)
Antigen Received (Based on Card or Recall)	164 (43.0)
**Inactivated poliovirus vaccine (IPV1)**	
Antigen Not Received	204 (53.5)
Antigen Received (Based on Card or Recall)	177 (46.5)
**At 9 Months**	**N = 198**
**Measles1**	
Antigen Not Received	79 (39.9)
Antigen Received (Based on Card or Recall)	119 (60.1)
**Inactivated poliovirus vaccine (IPV2)**	
Antigen Not Received	163 (82.3)
Antigen Received (Based on Card or Recall)	35 (17.7)
Typhoid	
Antigen Not Received	165 (83.3)
Antigen Received (Based on Card or Recall)	33 (16.7)
**At 15 Months**	**N = 126**
**Measles2**	
Antigen Not Received	50 (39.7)
Antigen Received (Based on Card or Recall)	76 (60.3)

Table S2: Baseline Characteristics of Children by Poor Clinical Outcome

	Total	Poor Clinical Outcome[¥]		Unadjusted			Adjusted		
		Yes	No						
	N=452	N=4	N=448						
	n (%)	n (%)	n (%)	OR	95% CI	P	OR	95% CI	P
**Child Characteristics**									
**Age category (in months)**									
02-11	293 (64.8)	3 (75.0)	290 (64.7)	1.63	(0.17,15.84)	0.672	-	-	-
12-59	159 (35.2)	1 (25.0)	158 (35.3)	1			-	-	-
**Sex**									
Male	273 (60.4)	4 (100.0)	269 (60.0)	-	-	-	-	-	-
Female	179 (39.6)	0 (0.0)	179 (40.0)	-	-	-	-	-	-
**Weight for Age Z-score**									
<=-3	137 (30.3)	2 (50.0)	135 (30.1)	3.05	(0.27,33.99)	0.364	1.266	(0.071,22.442)	0.872
-3 < WAZ <=-2	108 (23.9)	1 (25.0)	107 (23.9)	1.93	(0.12,31.08)	0.644	1.691	(0.094,30.292)	0.721
<-2	207 (45.8)	1 (25.0)	206 (46.0)	1			1		
**Height for Age Z-score**									
<=-3	119 (26.3)	1 (25.0)	118 (26.3)	1.03	(0.09,11.47)	0.981	-	-	-
-3 < HAZ <=-2	88 (19.5)	1 (25.0)	87 (19.4)	1.4	(0.13,15.6)	0.786	-	-	-
<-2	245 (54.2)	2 (50.0)	243 (54.2)	1			-	-	-
**Weight for Length Z-score**									
<-3	68 (15.0)	1 (25.0)	67 (15.0)	2.25	(0.2,25.14)	0.511	-	-	-
-3 <= WLZ <-2	81 (17.9)	1 (25.0)	80 (17.9)	1.88	(0.17,21.01)	0.608	-	-	-
>=-2	303 (67.0)	2 (50.0)	301 (67.2)	1			-	-	-
**Socio Economic Status**									
**Parent's Educated**									
Both Uneducated	332 (73.6)	2 (66.7)	330 (73.7)	0.07	(0.01,0.86)	0.037	0.032	(0.001,0.542)	0.018
Both Educated	36 (8.0)	0 (0.0)	36 (8.0)	1			1		
Only Father Educated	70 (15.5)	0 (0.0)	70 (15.6)	-	-	-	-	-	-
Only Mother Educated	13 (2.9)	1 (33.3)	12 (2.7)	-	-	-	-	-	-

Clinical Characteristics									
**Temperature Category**									
<37	46 (11.2)	1 (33.3)	45 (11.1)	3.84	(0.34,43.25)	0.276	-	-	-
37.0 - 38.9	348 (84.9)	2 (66.7)	346 (85.0)	1			-	-	-
>38.0 - 38.9	16 (3.9)	0 (0.0)	16 (3.9)	-	-	-	-	-	-
**Immunisation status**									
Fully immunized	70 (15.5)	0 (0.0)	70 (15.6)	-	-	-	1		
Partially immunized	305 (67.5)	3 (75.0)	302 (67.4)	1			3.183	(0.191,52.862)	0.419
Not immunized	77 (17.0)	1 (25.0)	76 (17.0)	1.33	(0.14,12.91)	0.809			
**Name of Medicine**									
Oral amoxicillin	282 (62.4)	1 (25.0)	281 (62.7)	1			-	-	-
Oral cefixime	114 (25.2)	3 (75.0)	111 (24.8)	7.6	(0.78,73.79)	0.081	-	-	-
Oral amoxicillin/clavulanic acid	25 (5.5)	0 (0.0)	25 (5.6)	-	-	-	-	-	-
Oral azithromycin	18 (4.0)	0 (0.0)	18 (4.0)	-	-	-	-	-	-
Others*	13 (2.9)	0 (0.0)	13 (2.9)	-	-	-	-	-	-

WAZ - Weight for age, HAZ - Height-for-age Z-score, WHZ - Weight-for-height z-score

¥Poor clinical outcome" indicates children who either died or were hospitalised between Day 1 and Day 15 of follow-up. Children not meeting these criteria were categorised as "No.

**Other medicines including Cefpodoxime, Cephalexin, Ciprofloxacin, Sulfamethoxazole-trimethoprim, Fosfomycin, and Linzolid*

OR; Odds Ratio, CI; confidence Interval

Adjusted for Weight-for-Age Z score, parental education, and immunisation status.

Table S3: Addendum Data on Amoxicillin Stockouts at the time of enrolment

Addendum Information

	Total
	N=246
**If the caregiver adhered to the advice, from where did they get antibiotics?**	
Antibiotics provided by the health facility free of cost	134 (54.5)
Antibiotics purchased by the caregiver	111 (45.1)
Any other, specify	1 (0.4)
**If a full course of antibiotics was not utilised / consumed, what was the reason?**	
Completed the course	134 (54.5)
Improved	90 (36.6)
No improvement	22 (8.9)
**Is the child on any treatment for chest indrawing pneumonia at the time of follow-up visit?**	
Yes	32 (13.0)
No	214 (87.0)
**Specify the type of treatment that the child is receiving at follow-up**	
Out-patient treatment	31 (97)
In-patient treatment	1 (3)
**If on OPD treatment at the time of follow-up, type of health facility/clinic/ health care provider giving treatment**	
Government health facility	5 (16)
Private health facility	24 (77)
Over the counter drug purchase /self-medication by the caregiver	2 (6)
**If on OPD treatment at the time of follow-up, was the child given antibiotics?**	
Yes	31 (100)
**If on OPD treatment at the time of follow-up, details of antibiotic: Name of medicine**	
Amoxil	2 (6)
Augmentin	1 (3)
Azithromycin	6 (19)
Cefalor	2 (6)
Cefixime	15 (48)
Clarithromycin	2 (6)
Cofex	1 (3)
Entamizole	1 (3)
Levofloxacin	1 (3)
**Is the child admitted in the hospital at the time of the visit?**	
yes	1 (3)
No	31 (97)
**If currently admitted at the time of follow-up visit, type of health facility/clinic**	
Government hospital	1 (100)
**Is the child admitted in the hospital at the time of the visit?**	
Yes	1 (3)
No	31 (97)
**If currently admitted at the time of follow-up visit, type of health facility/clinic**	
Government hospital	1 (100)

Table S4: Details of antibiotics prescribed at each health facility

Name of oral antibiotics given to children followed up (n=452)	Health Facility Location			Total
	BHU Dhabeji	RHC Varr	RHC Jungshahi	
	N=131	N=156	N=169	N=456
**Oral amoxicillin**	122 (93.1%)	132 (84.6%)	28 (16.6%)	282 (61.8%)
**Oral cefixime**	1 (0.8%)	17 (10.9%)	96 (56.8%)	114 (25.0%)
**Oral amoxicillin/clavulanic acid**	0 (0.0%)	1 (0.6%)	24 (14.2%)	25 (5.5%)
**Oral azithromycin**	1 (0.8%)	4 (2.6%)	13 (7.7%)	18 (3.9%)
**Others***	5 (3.8%)	0 (0.0%)	8 (4.7%)	13 (2.9%)
**Missing**[†]	2 (1.5%)	2 (1.3%)	0 (0.0%)	4 (0.9%)

BHU - Basic health unit, RHC - Rural health Centre

[*] Other medicines including Cefpodoxime, Cephalexin, Ciprofloxacin, Sulfamethoxazole-trimethoprim, Fosfomycin, and Linezolid
†Lost to follow-up
